# Validation of a Smartphone Image-Based Dietary Assessment Method for Pregnant Women

**DOI:** 10.3390/nu9010073

**Published:** 2017-01-18

**Authors:** Amy M. Ashman, Clare E. Collins, Leanne J. Brown, Kym M. Rae, Megan E. Rollo

**Affiliations:** 1 School of Health Sciences, Faculty of Health and Medicine, University of Newcastle, University Drive, Callaghan 2308, New South Wales, Australia; amy.ashman@uon.edu.au (A.M.A.); clare.collins@newcastle.edu.au (C.E.C.); 2 Priority Research Centre in Physical Activity and Nutrition, Faculty of Health and Medicine, University of Newcastle, University Drive, Callaghan 2308, New South Wales, Australia; 3 Gomeroi gaaynggal Centre, Faculty of Health and Medicine, University of Newcastle, 2/1 Hinkler Street, Tamworth 2340, New South Wales, Australia; kym.rae@newcastle.edu.au; 4 Department of Rural Health, Faculty of Health and Medicine, University of Newcastle, 114-148 Johnston Street, Tamworth 2340, New South Wales, Australia; leanne.brown@newcastle.edu.au; 5 Priority Research Centre in Reproduction, Faculty of Health and Medicine, University of Newcastle, University Drive, Callaghan 2308, New South Wales, Australia

**Keywords:** nutrition assessment, pregnancy, mHealth, image-based dietary records, Indigenous

## Abstract

Image-based dietary records could lower participant burden associated with traditional prospective methods of dietary assessment. They have been used in children, adolescents and adults, but have not been evaluated in pregnant women. The current study evaluated relative validity of the DietBytes image-based dietary assessment method for assessing energy and nutrient intakes. Pregnant women collected image-based dietary records (via a smartphone application) of all food, drinks and supplements consumed over three non-consecutive days. Intakes from the image-based method were compared to intakes collected from three 24-h recalls, taken on random days; once per week, in the weeks following the image-based record. Data were analyzed using nutrient analysis software. Agreement between methods was ascertained using Pearson correlations and Bland-Altman plots. Twenty-five women (27 recruited, one withdrew, one incomplete), median age 29 years, 15 primiparas, eight Aboriginal Australians, completed image-based records for analysis. Significant correlations between the two methods were observed for energy, macronutrients and fiber (*r* = 0.58–0.84, all *p* < 0.05), and for micronutrients both including (*r* = 0.47–0.94, all *p* < 0.05) and excluding (*r* = 0.40–0.85, all *p* < 0.05) supplements in the analysis. Bland-Altman plots confirmed acceptable agreement with no systematic bias. The DietBytes method demonstrated acceptable relative validity for assessment of nutrient intakes of pregnant women.

## 1. Introduction

Pregnant women have unique nutrition requirements for growth and development of the fetus, and health of both mother and child [1,2,3]. However, in Australia women of childbearing age may be at risk of not meeting these recommendations during this period [4,5,6]. In order to assist pregnant women with optimizing their dietary intake it is imperative to first ascertain what they are currently eating and drinking. Dietitians in all areas of practice depend on validated, reliable tools for the assessment of dietary intake [7]. Self-reported dietary intake is a feasible and practical way to establish intake in both clinical practice and research settings, although there are challenges associated with this method. Diet is complex, affected by food availability and personal preferences, and intake can vary on a day-to-day basis. Dietary assessment methods may be susceptible to bias, including under-reporting of energy intake [8,9]. Prospective methods, including weighed and estimated food records, require the reporting of all food and drinks consumed. Weighing or estimating all foods may be burdensome for individuals, requiring high levels of motivation to keep accurate records [10], and accuracy can decrease if records need to be kept for more than four days [11]. The process of keeping records may result in changes to usual intake [12,13], and the act of keeping the records themselves requires a degree of numeracy and/or literacy skill from individuals [14,15].

Image-based dietary records are emerging as a novel method for dietary assessment, and may be able to address some of the participant burden associated with traditional prospective methods such as weighed records. Their use involves capturing images of food and drinks consumed in order to support paper dietary records, or to act as standalone dietary records. Images can be transferred to a dietitian or other trained individual for analysis and interpretation, shifting the onus of estimating portion size from the individual to the dietitian [16]. Advancements in smartphone technology have resulted in a unique platform for the capture and relaying of image-based dietary records in real time. Smartphone ownership is prevalent, with 77% of Australian adults owning smartphones, and ownership is on the rise [17]. Smartphone features such as internet connectivity and built-in cameras support the use of this platform for collection of image-based dietary records. While the use of image-based or image-assisted dietary assessment has been explored in populations of healthy adults [18,19,20,21], children and adolescents [22,23,24], overweight and obese adults [20], and adults with type 2 diabetes [16,25], their use has not been evaluated in pregnant women, warranting further investigation. 

Importantly, the use of novel dietary assessment methods in new population groups requires validation in order for them to be utilized in a variety of research and clinical practice settings. The current study therefore reports on the evaluation of the DietBytes image-based dietary assessment method in a group of pregnant women, with a focus on Indigenous women. In the Diet Bytes and Baby Bumps study (DBBB) pregnant women used a smartphone application (app) to capture three-day image-based dietary records (the DietBytes method). The study aimed to: (1) assess the relative validity of image-based dietary records for assessment of intake of Indigenous and non-Indigenous Australian pregnant women, against three 24-h (24-R) food recalls; (2) assess the inter-rater reliability between two independent dietitians in assessing 3-day image-based dietary records and 24-R recalls in a sub-sample of participants (*n* = 10); (3) assess the quality of image-based dietary records and voice/text description for analysis; (4) assess the perceived usability and acceptability of the image-based dietary assessment method by the pregnant women.

## 2. Materials and Methods

### 2.1. Ethics

DBBB was approved by the following ethics committees; Aboriginal Health and Medical Research Council Ethics Committee (Reference No. 962/13), Hunter New England Human Research Ethics Committee (HREC Reference No. 13/06/19/4.04) and the University of Newcastle Human Research Ethics Committee (Reference No. H-2013-0185). 

### 2.2. Eligibility

Women were eligible for inclusion if they met the following criteria: ≤24 weeks gestation, aged ≥18 years, no current medical conditions (including gestational diabetes), ownership of or access to a smartphone capable of using the freely downloadable app Evernote^®^ for smartphones and computers (Mobile and desktop app software, 2016 Evernote Corporation, Redwood City, CA, USA), and willingness to attend two in-person sessions. All participants gave written, informed consent.

### 2.3. Recruitment and Setting

Recruitment for DBBB was conducted in Tamworth, a regional inland town in New South Wales (NSW), and Newcastle, the second largest city in NSW, Australia. Participants were recruited at antenatal clinics by members of the research team at both sites. In addition, the study was advertised via promotional fliers at hospital antenatal and general practitioner clinics and at the University of Newcastle campus, as well as through social media. In Tamworth, members of the research team, including an Indigenous research assistant, recruited through the Gomeroi gaaynggal Centre [26]. While no specific sample calculation was performed, a target of 25 pregnant women with adequate image-based records was set given the substantial participant burden on pregnant women to collect the data.

### 2.4. DietBytes Dietary Assessment Method

**Figure 1:**
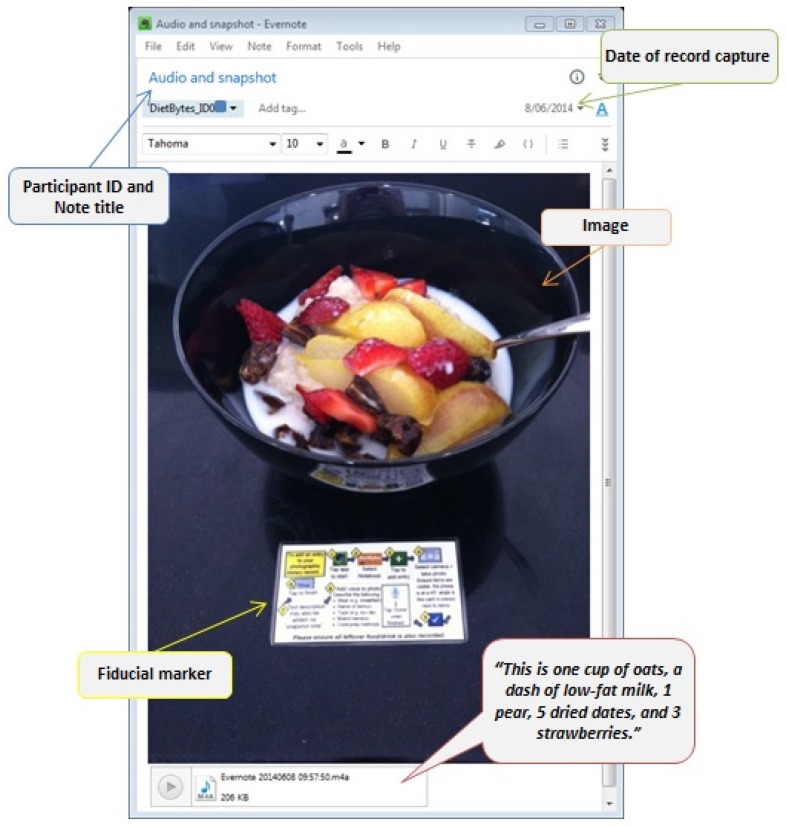
Example of an eating occasion recorded in the image-based dietary records in the Diet Bytes and Baby Bumps study, including supporting voice description, fiducial marker, and date and title of record.

Dietary assessment in DBBB was modelled on methods previously used in adults with Type 2 diabetes [16,25]. In week one of the study, participants used the Evernote^®^ app to record all eating and drinking occasions (including vitamin and mineral supplement use) for three non-consecutive days, including a weekend day (the DietBytes method). Records consisted of taking a phone image of the consumed item(s) placed next to a fiducial marker (reference object of known dimensions). Text and/or voice descriptions were added to the image to support the identification of items in the image, and included detailing brands, types, and cooking methods of foods consumed, where applicable (Figure 1). Participants were instructed to record images of all food and drink leftover, and any second servings consumed. No prior familiarity with the Evernote^®^ app, or experience with recording dietary intake, was required. Training was provided in the first in-person session (week one) and participants created a practice record. Records could only be viewed by the participant and members of the research team who had access to the DietBytes Evernote^®^ account. Settings on the app were selected so that records could only be shared with the research team over a Wi-Fi connection and/or were uploaded to the DietBytes Evernote^®^ account during the second in-person session (week two). Participants were encouraged to label their records (e.g., Snack), however the Evernote^®^ app automatically notes the date and time a record is made, which assisted the research team with establishing when items were consumed.

### 2.5. 24-h Recall (24-R)

In weeks two, three and four, participants were asked to complete a dietitian-administered 24-R (one per week). Diet recalls in week two were conducted at the in-person study session, while the recalls in week three and week four were collected over the telephone. The three collection days were varied across the week and consisted of one weekend day. A multiple-pass method was used: (1) participants reported a quick list of all items consumed in the previous 24-h period; (2) followed by a checklist for forgotten foods; and (3) probing for detail (i.e., amounts, type, cooking/preparation methods) on foods listed (based on standardized protocols for multiple-pass 24-R) and review [27,28]. To assist with estimating portion size of foods consumed, participants were given a visual aid (booklet), the Dietary Estimation and Assessment Tool (DEAT) [16,25]. The DEAT consisted of images of foods and drinks in varying portion sizes, serving vessels, amorphous mounds and geometric shapes and was based on similar food model booklets [29,30]. Participants used the DEAT to quantify amounts of foods and drinks consumed, by indicating which portion size they consumed.

### 2.6. Nutrient Analysis

For the current study, energy, macronutrient and micronutrient intakes from the image-based dietary records and 24-R were assessed using FoodWorks^®^ (Xyris Software, Pty Ltd., Brisbane, Queensland, Australia) nutrient composition software, with AUSNUT 2007 [31] selected as the nutrient composition table (“foods”, “brands”, and “supplements” selected). A protocol was developed to standardize entries into FoodWorks^®^, including common assumptions made (for example, the ‘not further specified’ option was used when detail was lacking; specific brands were not chosen unless they were explicitly stated). Two portion size estimation aids were used to assist in the quantification of items contained in image-based dietary records. In addition to the DEAT (where quantities of foods and serving vessels were displayed), a separate visual guide consisting of 80 images of a variety of food and drinks, photographed in serving size amounts recommended in the Australian Dietary Guidelines [3] was developed. One dietitian performed the analysis of the image-based dietary records and 24-R for all (*n* = 25) participants. In order to determine the inter-rater reliability, a second dietitian analysed the image and 24-R records for a sub-sample of 10 participants.

### 2.7. Quality Assessment of Image-Based Dietary Records

The quality of the image-based dietary records was established by examining records against the following pre-defined set of criteria using a “yes” or “no” response. Each entry (eating and/or drinking occasion) was evaluated against a checklist for the following components: an image; text description; and voice record description. 

### 2.8. Surveys

Participants completed online surveys over the course of the study. The week one survey asked questions on demographics, usual use of smartphones, and on nutrition information received prior to study enrolment. The week two survey asked about participants’ perceived usability and acceptability of the DietBytes method of dietary assessment using Likert scale, multiple-choice, and yes/no responses; and open-ended questions providing an opportunity to record qualitative responses.

### 2.9. Statistical Methods

Variables were assessed for normality of distribution graphically and via the Shapiro-Wilk test. Inter-rater reliability between two independent dietitians who assessed the image-based dietary records and 24-R in FoodWorks^®^ was assessed via intra-class correlation coefficients for energy and nutrient intakes of the sub-sample of participants (*n* = 10). Relative validity was assessed from one dietitian’s analysis of the image-based dietary records compared with the 24-R recalls for all participants (*n* = 25). The strength of linear relationships between the two methods was evaluated using Pearson correlations, one-sample *t*-tests exploring differences between the two measures, and agreement assessment using Bland-Altman plots and to assess any systematic bias between methods. Descriptive statistics and frequencies are provided for demographic data. Analyses were performed using IBM SPSS statistical software (Version 23.0, IMB Corp., Armonk, NY, USA). Statistical significance was set at *p* ≤ 0.05. Results from the quality assessment of the image-records are reported as counts and percentages. A general inductive approach was used to analyze the qualitative responses to the open-ended survey questions about usability and acceptability (week two survey) [32]. This approach involved close reading of the qualitative text, creation of categories, coding of data into categories, revision and refinement of categories. The categories capture key aspects of themes present within the raw data.

## 3. Results

### 3.1. Participant Characteristics

Twenty-seven women enrolled in the DBBB study, with one withdrawal. Of the 26 participants who completed DBBB, one participant completed two days of the image-based dietary record and one completed one day. The participant completing only one day was excluded from all analyses. Therefore results reported here are for *n* = 25 participants. Of these 25 participants, 17 were recruited from the Tamworth recruitment sites, and eight from the Newcastle sites; eight identified as Indigenous Australians (all identified as Aboriginal), and 17 as non-Indigenous. The median age of participants at recruitment was 28.8 years (range: 20.4–50.4 years). Gestation at the time of recruitment ranged from 6–24 weeks, with four participants in their first trimester of pregnancy and 21 in the second trimester. Twelve participants (48%) had measured or kept a record of their diet previously (e.g., for previous health condition or for a previous research study) and the remaining participants had not kept a dietary record before participation in DBBB. The most commonly used apps that participants used on their smartphones were social media apps (*n* = 32 responses), games (*n* = 12), banking (*n* = 9), baby/pregnancy-related app (*n* = 9) and emails (*n* = 5). Further characteristics of DBBB study participants and smartphone uses are summarized in Table 1. 

### 3.2. Relative Validity of the DietBytes Method

Results of Pearson correlations, mean difference between methods, and one-sample *t*-tests are summarized in Table 2. There was no significant difference between dietary assessment methods for intakes of energy (mean difference 517 ± 1461 kJ/day, *t*^(*df*)^ = 1.77^(24)^, *p* = 0.089), or carbohydrate and protein intakes, although the mean difference of 7.8 ± 18.7 g fat/day was statistically significant (*t*^(*df*)^ = 2.08^(24)^, *p* = 0.049). There was no significant difference for daily micronutrients iron, iodine, folate, zinc and calcium, either with or without supplements included in the analysis.

**Figure 2:**
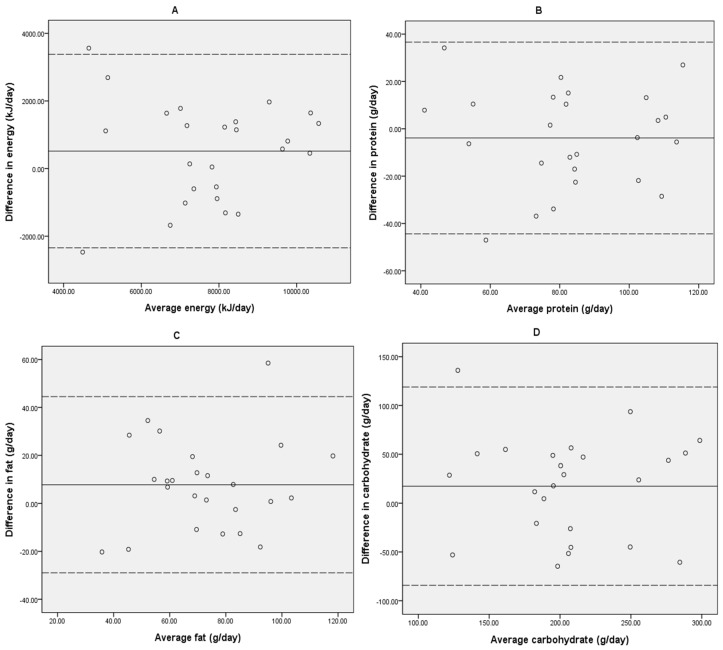
Bland-Altman plots showing mean difference (24-R − DietBytes, solid line) vs. mean intakes ((24-R + DietBytes)/2) between the DietBytes image-based dietary records and 24-R nutrient intakes, and two standard deviations of the difference (limits of agreement, dotted lines), for the following: (**A**) Energy (kilojoules per day); (**B**) Protein (grams per day); (**C**) Fat (grams per day); (**D**) Carbohydrate (grams per day).

Bland-Altman plots were constructed for energy, macronutrients, and micronutrients (see Figure 2 for plots of energy and macronutrients). Bland-Altman plots comparing mean intakes versus the difference between the image-based dietary records and 24-R methods for daily energy and macronutrient intakes indicates the majority of values were within the acceptable limits of agreement.

### 3.3. Inter-Rater Reliability

Results of the nutrient analysis performed by two dietitians using the two dietary assessment methods, for a sub-sample of *n* = 10 participants are summarized in Table 3. Intra-class correlation coefficients between the two dietitians for the analysis of the image-based dietary records was 0.929 (*p* < 0.001) for energy, 0.865–0.932 (all *p* < 0.05) for macronutrients carbohydrate, protein, and fat; and ranged from 0.794–0.988 (all *p* < 0.05) for selected key micronutrients (folate, iron, iodine, calcium and zinc). Intra-class correlation coefficients between the two dietitians for the analysis of the 24-Rs was 0.973 (*p* < 0.001) for energy, 0.952–0.978 (all *p* < 0.001) for macronutrients, and 0.921–0.989 (all *p* ≤ 0.001) for the aforementioned micronutrients.

### 3.4. Quality Assessment of the Image-Based Dietary Record Entries

There were a total of 517 record entries (recorded eating and/or drinking occasions, consisting of image and/or voice record and/or text description) for the 25 participants (20.7 ± 9.2 entries per participant). The majority of entries included an image (*n* = 496, 96%), over half of the entries included text description providing additional details (*n* = 312, 60%), and around one third of entries included voice description to provide additional detail (*n* = 158, 31%). A small proportion of entries contained an image, text description, and a voice record (*n* = 15, 3%). Further details of results from the quality assessment are displayed in Table 4.

### 3.5. Perceived Usability and Acceptability of Using the DietBytes Method

In the week two survey, participants (*n* = 25) were asked about the usability and acceptability of the dietary assessment methods used in DBBB. Overall, 22 participants (88%) said they would be willing to use the DietBytes method again, including all Aboriginal participants. Of these women, nine reported they would use the image-based method again for up to one week, with five expressing they would use the method for one month or more, while others would use it for three days or less (*n* = 8). The majority of participants (*n* = 21, 84%) rated their satisfaction with the Evernote^®^ app as ‘satisfied’ (*n* = 15) or ‘very satisfied’ (*n* = 6). Further quantitative responses to the week two survey are summarized in Table 5. 

The qualitative data participants provided on the acceptability and usability of the DietBytes method revealed two key themes: (i) Process and perceptions of using the image-based dietary record; and (ii) Changes to dietary intake due to increased awareness and external influences. 

Under the first theme ‘Process and perceptions of using the image-based dietary record’ participants commented about the process of using the image based dietary record, and external perceptions of the DietBytes method. Participants commented that keeping the dietary record involved memory, i.e., remembering (or forgetting) to record items, or remembering to place the fiducial marker in the images. This was cited as a reason for not recording all food and drink items, and also as something participants found to be a barrier to completing the record.

“It was often difficult to remember to take the pictures and to put the prompt card in the pictures.”

—Age 27, first baby, non-Indigenous participant

Some participants also reported that having to keep the dietary record could be inconvenient, e.g., having to have their phone with them before they could eat, or avoiding shared meals that were complicated to record. Some felt self-conscious or embarrassed to record intake using the image-based dietary record, as illustrated in the following survey quote:

“I didn’t like to eat out during this time due to not being comfortable photographing my food in front of others.”

—Age 33, first baby, Aboriginal participant

In particular, there were some negative responses to using the voice record in public:

“[I] was more self-conscious to use the voice recording if other people were around so tended to use the text instead.”

—Age 35, first baby, non-Indigenous participant

Some participants commented on the process being “quick” and “easy” to use, and that using the DietBytes method was preferable to other dietary assessment methods:

“It didn’t require me to measure and log each ingredient, something which has discouraged me from using food diaries in the past.”

—Age 30, first baby, non-Indigenous participant

Under the second theme, a sub-theme arose of ‘increasing awareness’: participants indicated that keeping an image-based dietary record increased their awareness of their dietary intake. Some participants commented that the act of collecting the image-based record had a positive influence on their eating behaviors, including choosing healthier food options:

“Seeing pictures of dietary intake is a good motivator to make good choices!”

—Age 27, first baby, non-Indigenous participant

Other changes to intake as a result of having to keep the dietary record, included “[choosing] foods that were easier to record” eating less; and “[a] combination of eating more at some meals [and] less at others”; and not eating out as often. Other influences on participants’ dietary intake included the impact of someone else being able to see what they were eating:

“[I] used the fact someone else would see what I ate to break a bad habit that formed in the last month of having something sweet at 3:00 p.m. Didn’t want to have it any more so used it for self-motivation to break habit.”

—Age 35, first baby, non-Indigenous participant

Some negative responses arose from the influence of others observing participants’ dietary intake (including the dietitian performing the analysis, and family members). One participant reported guilty feelings around taking images of sweets, biscuits and chocolates. Another commented:

“With family around me adding their own input for anything that I had forgotten I found it very distracting.”

—Age 36, second baby, Aboriginal participant

## 4. Discussion

The first aim of the current study was to establish the relative validity of the image-based records against the 24-R. Pearson correlations comparing estimated nutrient intakes between the two methods were moderate to substantial for energy, macronutrients and fiber (*r* = 0.58–0.84, all *p* < 0.05), and for micronutrients both with supplement use included (*r* = 0.47–0.94, all *p* < 0.05) and without supplement use included (*r* = 0.40–0.85, all *p* < 0.05). In addition, there were no significant mean differences in nutrients between the two dietary assessment methods, with the exception of total fat (borderline at *p* = 0.049) and saturated fat (*p* = 0.008). However, mean differences were small and not clinically important for any nutrient. The 95% Confidence Intervals (limits of agreement) are relatively wide in the Bland-Altman plots for energy and macronutrients, which shows variability between methods for individuals. However, most data points are within the limits of agreement with only one or two outliers, and the pattern of data distribution in the Bland-Altman plots does not indicate evidence of systematic bias at high or low intakes. The second aim was to establish the inter-rater reliability between two dietitians for assessing the image-based dietary records and 24-R. Intra-class correlation coefficient test statistics for macronutrients and major micronutrients that are particularly important during pregnancy (iron, calcium, zinc, iodine and folate) were in the excellent range (0.75–1 is considered excellent agreement) [33] and one dietitian subsequently analyzed records for all (*n* = 25) participants.

The results of DBBB are very encouraging, and demonstrate acceptable validity of the DietBytes method for dietary assessment of pregnant women. In a previous study in Japan by Wang and colleagues, *n* = 20 female college students studying food and nutrition recorded one day of dietary intake through both a weighed food record and by capturing images of the same meals. Images were captured via handheld personal digital assistant with camera and mobile ‘phone card (the Wellnavi method). Resulting Spearman’s rank correlation coefficients of *r* = 0.46–0.93 (median *r* = 0.77) were deemed acceptable for demonstrating the use of the image-based dietary record [21]. In a follow up study of *n* = 28 participants there was a median correlation coefficient of *r* = 0.066 for nutrients between the two dietary assessment methods, with 57% of participants reporting the Wellnavi method as less burdensome and less time-consuming compared to weighed food records or 24-R [34]. This suggests that the results of the current DBBB study are comparable with this previous study examining the use of image-based dietary records in young women, and have provided support for their use. 

The current study sought to assess the quality of the image-based dietary records, in order to establish if these could feasibly be analyzed. While two-thirds of the record entries included text description (*n* = 312, 60%), only one third included voice description (*n* = 158, 31%). This was reflected in survey responses, with only 20% (*n* = 5) of participants favoring the voice description over text. Participants reported feeling self-conscious or embarrassed when using the voice record in public. Although the voice records can provide more detailed description to support images, this is an important issue to acknowledge. Of interest was that for 89% of entries containing an image, the image alone was sufficient to quantify items. A recommendation for future use of the DietBytes method is to reinforce to participants that records can be amended at a later time point, and additional description, whether text or voice, can be added to entries when in a quiet and private space. 

The DietBytes method was well-received by pregnant women in this study; all but one reported that the Evernote^®^ app was easy to use, 84% (*n* = 21) were satisfied with the app, and 88% (*n* = 22) stated that they would use the image-based dietary record method again. Importantly, all Aboriginal participants were willing to use the method again. Aboriginal and Torres Strait Islander Australian women face socio-economic barriers to nutrition, including disadvantages in education, employment and income. In addition, there are geographic limitations to accessing nutritious foods in rural and remote areas [35], where nearly two thirds (65%) of Australia’s Indigenous Australians reside [36]. Indigenous Australian women may therefore be at higher risk for food insecurity [37] and have dietary intakes that differ from their non-Indigenous counterparts. That the DietBytes method was well-received may be a consideration for researchers working in the field of Aboriginal nutrition, as a potential method for dietary assessment which has demonstrated acceptability by this group of pregnant women.

The DietBytes method may have been particularly acceptable for this cohort of women of childbearing age, as 92% (*n* = 23) of participants reported that they use their phones for taking, sending, and uploading photographs at the time of recruitment. Our previous study demonstrated that providing feedback on the image-based dietary records via the smartphone in combination with consultation with a dietitian was well-received by participants [38]. The majority of women in the current study (*n* = 18, 72%) indicated they would use the DietBytes method again to obtain feedback from a dietitian, but interestingly, over half (*n* = 16, 64%) would use it for their own feedback or tracking of their diet (i.e., self-monitoring). Previous research with young women demonstrated that computer and smartphone food records were as accurate as paper-based records for dietary self-monitoring, and that these methods were preferred over the paper-based records [39].

A sub-theme that arose from survey responses was that the act of keeping a dietary record increased participants’ awareness of the food they ate. The findings of DBBB are mirrored in other studies where participants have reported increased awareness of foods or portion sizes consumed as a result of capturing images of food intake [40]. Participation in the DietBytes study may have created a teachable moment, by motivating women to consider dietary changes, and previous research has demonstrated that pregnancy is a time period when women may gain an increased awareness of their dietary intake [41] and be more receptive towards engaging in healthy eating behaviors [42]. The downside of this phenomenon is that having to record dietary intake may cause people to change their usual eating behaviors: this is a common limitation of dietary assessment methods and is not unique to the DietBytes method [12,13]. However, this was unlikely to have had a major influence on the validity of the DietBytes method, as only a small proportion of participants reported that they changed the type (*n* = 7, 28%), frequency (*n* = 6, 24%) or amount (*n* = 3, 12%) of food they ate when they used the DietBytes method, with even fewer reporting changes to where they ate, who they ate with, or to their cooking habits. This is reflected in the high agreement between the DietBytes method and 24-R.

The two methods of dietary assessment used (DietBytes and the 24-R) were chosen as they had theoretical errors independent of one another: DietBytes is a prospective method that puts the onus of portion size estimation on the dietitian, and the 24-R method is retrospective and requires participants to estimate and report portion sizes. Choosing two different methods reduces the chance of correlations between nutrient intakes due to similar errors, however both methods have the potential for participants to misreport dietary intake [43]. 

There are limitations to the current study that should be acknowledged. DBBB participants may not be representative of all pregnant women in Australia. All participants were born in Australia (Australia-wide, 28.2% of the resident population were born overseas [44]), and all spoke only English at home; so language did not act as a barrier to study participation or accessing antenatal care in general. Over half (*n* = 14, 56%) of participants held a university or higher university degree, compared with 29% of Australian women aged 15–64 years [45], which is likely attributed to the fact that a major source of recruiting was via a university campus. DBBB excluded women who did not own a smartphone, and depended on women having their smartphone on hand during eating and drinking occasions. Therefore women who did not have regular access to a smartphone could not participate. While smartphone ownership is high in Australia (77%) [17], the study design may have excluded economically vulnerable women. However, there was a broad distribution of income represented. Finally, previous research has shown that three days may be adequate for establishing mean energy intakes of groups, however may not be a long enough duration to accurately measure intake of macro- and micro-nutrients [46]. More days of recording may therefore have been required. Eight of the 22 participants in DBBB who reported that they would be willing to use the DietBytes method again would use it for three days or less. However, the remaining 14 participants would be willing to use the method for longer recording times, the majority of whom reported a maximum of one week. Therefore, there is potential for future research to explore the use of the DietBytes method over longer recording periods.

## 5. Conclusions

The DietBytes method of image-based dietary assessment demonstrated acceptable relative validity for establishing energy and nutrient intakes of pregnant Aboriginal and non-Aboriginal Australian women. The use of image-based dietary records was well-received by participants, the majority of whom would be willing to use the method again. The DietBytes method of dietary assessment via image-based records may therefore be a useful and feasible way for dietitians in research or practice settings to establish dietary intakes of pregnant women.

## Figures and Tables

**Table 1:** Demographic characteristics of participants in the Diet Bytes and Baby Bumps study (*n* = 25).

Characteristic	*n* (%)
*Aboriginal or Torres Strait Islander origin*	8 (32)
*Born in Australia*	25 (100)
*Speaks only English at home*	25 (100)
*Currently smokes tobacco products*	4 (16)
*Ever had to measure or keep a record of diet or been asked to recall foods eaten*	12 (48)
*Type of smartphone currently used:*	
iPhone	18 (72)
Android	7 (28)
*Highest qualification completed:*	
No formal qualifications	1 (4)
School certificate (year 10 or equivalent)	1 (4)
Higher school certificate (year 12 or equivalent)	3 (12)
Certificate/ Diploma (e.g., childcare, technician)	6 (24)
University Degree	6 (24)
Higher University Degree	8 (32)
*Present marital status:*	
Never married	2 (8)
Defacto	8 (32)
Married	14 (56)
Separated, but not divorced	1 (4)
*Number of children:*	
This will be my first baby	15 (60)
1	4 (16)
2	3 (12)
≥3	3 (12)
*Average annual gross (before tax) household income*	
No income	0 (0)
$1–$31,199	0 (0)
$31,200–51,999	2 (8)
$52,000–77,999	6 (24)
$78,000–103,999	2 (8)
$104,000 or more	10 (40)
Don’t know	2 (8)
No response	3 (12)
*How do you manage on the income you have available?*	
It is easy	4 (16)
It is not too bad	12 (48)
It is difficult some of the time	7 (28)
It is difficult all of the time	1 (4)
It is impossible	1 (4)
*Smartphone activities*	
Sending SMS (text messages)	25 (100)
Receiving SMS (text messages)	24 (96)
Making voice calls	23 (92)
Making video calls	11 (44)
Taking photos	23 (92)
Sending and/or uploading photos	23 (92)
Taking videos	15 (60)
Sending and/or uploading videos	11 (44)
Searching or browsing the internet	24 (96)
Directors, maps and/or GPS functions	22 (88)
Taking notes	20 (80)
Playing games	13 (52)
Calendar or diary function	17 (68)
Playing music	18 (72)
Making voice recordings	4 (16)
Using apps	21 (84)

**Table 2:** Comparison of energy and nutrient intake between the DietBytes image-based dietary records and 24-h food recall methods (*n* = 25).

Nutrient	Method	Mean ± SD ^1^ Intake ^2^	Pearson Correlation between Methods	Mean Difference ^3^ ± SD	One-Sample *t*-Test (DF ^4^), *p*
Energy (kJ/day)	DBBB ^5^	7503 ± 1864	0.696 (*p* < 0.001)	517 ± 1461	1.77 (24), *p* = 0.089
	24-R ^6^	8020 ± 1884			
Protein (g/day)	DBBB	85.4 ± 23.8	0.619 (*p* = 0.001)	−3.9 ± 20.7	−0.94 (24), *p* = 0.355
	24-R	81.5 ± 23.6			
Fat, total (g/day)	DBBB	69.2 ± 21.5	0.654 (*p* < 0.001)	7.8 ± 18.7	2.08 (24), *p* = 0.049
	24-R	77.0 ± 23.4			
Fat, saturated (g/day)	DBBB	26.7 ± 8.1	0.745 (*p* < 0.001)	4.8 ± 8.3	2.901 (24), *p* = 0.008
	24-R	31.5 ± 12.4			
Carbohydrate (g/day)	DBBB	198.1 ± 57.6	0.580 (*p* = 0.002)	17.4 ± 51.8	1.68 (24), *p* = 0.107
	24-R	215.5 ± 55.4			
Fiber (g/day)	DBBB	22.2 ± 8.7	0.844 (*p* < 0.001)	0.6 ± 4.8	0.66 (24), *p* = 0.516
	24-R	22.8 ± 8.4			
**Supplements included: Food and supplements**					
Iron (mg/day)	DBBB	19.1 ± 16.7	0.622 (*p* = 0.001)	4.4 ± 17.5	1.25 (24), *p* = 0.224
	24-R	23.5 ± 22.1			
Vitamin C (mg/day)	DBBB	156.3 ± 180.8	0.549 (*p* = 0.004)	15.0 ± 151.6	0.50 (24), *p* = 0.624
	24-R	171.3 ± 112.3			
Folate ^7^ (µg/day)	DBBB	1210.6 ± 1693.2	0.937 (*p* < 0.001)	40.3 ± 735.5	0.2 7(24), *p* = 0.787
	24-R	1250.8 ± 1149.7			
Zinc (mg/day)	DBBB	15.8 ± 7.6	0.805 (*p* < 0.001)	0.5 ± 4.5	0.51 (24), *p* = 0.616
	24-R	16.3 ± 6.6			
Iodine (mg/day)	DBBB	198.3 ± 126.9	0.669 (*p* < 0.001)	18.3 ± 97.8	0.94 (24), *p* = 0.359
	24-R	216.6 ± 110.6			
Calcium (mg/day)	DBBB	875.9 ± 351.8	0.473 (*p* = 0.017)	−13.0 ± 324.2	−0.20 (24), *p* = 0.843
	24-R	862.9 ± 261.0			
Vitamin D (µg/day)	DBBB	7.6 ± 9.5	0.870 (*p* < 0.001)	−0.3 ± 4.8	−0.31 (24), *p* = 0.756
	24-R	7.3 ± 7.3			
Vitamin E (mg/day)	DBBB	11.5 ± 8.7	0.725 (*p* < 0.001)	1.2 ± 6.4	0.95 (24), *p* = 0.354
	24-R	12.7 ± 8.5			
Sodium (mg/day)	DBBB	2269.9 ± 825.6	0.687 (*p* < 0.001)	178.9 ± 683.5	1.31 (24), *p* = 0.203
	24-R	2448.7 ± 894.2			
Potassium (mg/day)	DBBB	2848.2 ± 813.1	0.659 (*p* < 0.001)	209.1 ± 722.0	1.45 (24), *p* = 0.161
	24-R	3057.3 ± 919.1			
Magnesium (mg/day)	DBBB	345.9 ± 149.0	0.842 (*p* < 0.001)	−1.76 ± 80.4	−0.109 (24), *p* = 0.914
	24-R	344.2 ± 121.5			
**Supplements excluded: Food only**					
Iron (mg/day)	DBBB	11.5 ± 4.0	0.562 (*p* = 0.003)	−0.24 ± 3.5	−0.341 (24), *p* = 0.736
	24-R	11.3 ± 3.4			
Vitamin C (mg/day)	DBBB	109.7 ± 70.5	0.502 (*p* = 0.011)	21.2 ± 87.8	1.209 (24), *p* = 0.238
	24-R	131.0 ± 98.5			
Folate ^7^ (µg/day)	DBBB	487.4 ± 286.2	0.404 (*p* = 0.045)	40.0 ± 279.9	0.714 (24), *p* = 0.482
	24-R	527.3 ± 214.5			
Zinc (mg/day)	DBBB	10.7 ± 3.1	0.513 (*p* = 0.009)	0.1 ± 2.8	0.103 (24), *p* = 0.918
	24-R	10.7 ± 2.5			
Iodine (mg/day)	DBBB	113.0 ± 51.4	0.575 (*p* = 0.003)	9.4 ± 44.3	1.055 (24), *p* = 0.302
	24-R	122.4 ± 43.6			
Calcium (mg/day)	DBBB	812.7 ± 310.7	0.466 (*p* = 0.019)	−0.6 ± 297.5	−0.010 (24), *p* = 0.992
	24-R	812.1 ± 258.5			
Vitamin D (µg/day)	DBBB	2.8 ± 1.3	0.615 (*p* = 0.001)	0.3 ± 1.5	0.979 (24), *p* = 0.337
	24-R	3.1 ± 1.9			
Vitamin E (mg/day)	DBBB	8.9 ± 5.5	0.766 (*p* < 0.001)	−0.4 ± 3.5	−0.515 (24), *p* = 0.611
	24-R	8.6 ± 4.3			
Sodium (mg/day)	DBBB	2269.8 ± 825.7	0.687 (*p* < 0.001)	178.8 ± 683.6	1.308 (24), *p* = 0.203
	24-R	2448.5 ± 894.2			
Potassium (mg/day)	DBBB	2844.5 ± 806.1	0.652 (*p* < 0.001)	209.6 ± 722.5	1.450 (24), *p* = 0.160
	24-R	3054.0 ± 911.2			
Magnesium (mg/day)	DBBB	318.1 ± 101.8	0.846 (*p* < 0.001)	−4.3 ± 56.6	−0.382 (24), *p* = 0.706
	24-R	313.8 ± 102.4			

^1^ SD Standard Deviation; ^2^ Mean (±SD) of three-day records for each method as assessed by Dietitian 1; ^3^ Mean difference (24-h recall intake − image record intake) calculated for each participant; ^4^ Degrees of freedom; ^5^ DBBB Analysis based on Diet Bytes & Baby Bumps image-based dietary records; ^6^ 24-R Analysis based on 24-h recall; ^7^ Folate as dietary folate equivalents.

**Table 3:** Inter-rater reliability of energy and nutrient intake from the Diet Bytes and Baby Bumps image-based dietary records and 24-h recalls between two dietitians (for *n* = 10 participants).

Nutrient	Method	Mean ± SD ^1^ Intake as Assessed by Each Dietitian		ICC ^2^ (95% CI) between Dietitians 1 & 2	*p*
		Dietitian 1	Dietitian 2		
Energy (kJ/day)	DBBB ^3^	7665 ± 1795	7786 ± 2654	0.929 (0.710–0.982)	<0.001
	24-R ^4^	7966 ± 2387	7728 ± 2539	0.973 (0.897–0.993)	<0.001
Protein (g/day)	DBBB	86.6 ± 19.0	90.7 ± 29.3	0.865 (0.471–0.966)	0.004
	24-R	79.5 ± 24.1	76.7 ± 25.4	0.978 (0.915–0.994)	<0.001
Fat, total (g/day)	DBBB	75.2 ± 21.9	78.7 ± 29.1	0.932 (0.738–0.983)	<0.001
	24-R	77.6 ± 28.0	71.0 ± 29.9	0.952 (0.790–0.988)	<0.001
Fat, saturated (g/day)	DBBB	28.9 ± 8.4	30.1 ± 12.2	0.886 (0.544–0.972)	0.002
	24-R	34.2 ± 13.7	31.1 ± 14.0	0.949 (0.786–0.987)	<0.001
Carbohydrate (g/day)	DBBB	193.9 ± 48.3	189.6 ± 73.1	0.930 (0.718–0.983)	<0.001
	24-R	213.4 ± 68.2	217.0 ± 69.7	0.975 (0.904–0.994)	<0.001
Fiber (g/day)	DBBB	20.1 ± 8.3	19.5 ± 6.9	0.923 (0.694–0.981)	<0.001
	24-R	21.5 ± 7.5	20.6 ± 7.0	0.983 (0.929–0.996)	<0.001
Iron (mg/day)	DBBB	12.2 ± 3.5	12.1 ± 3.3	0.810 (0.185–0.954)	0.014
	24-R	11.9 ± 4.1	11.4 ± 4.1	0.977 (0.912–0.994)	<0.001
Vitamin C (mg/day)	DBBB	96.3 ± 57.8	96.6 ± 89.0	0.893 (0.551–0.974)	0.002
	24-R	130.0 ± 80.2	117.9 ± 59.6	0.945 (0.793–0.986)	<0.001
Folate ^5^ (µg/day)	DBBB	644.2 ± 546.6	676.9 ± 400.1	0.954 (0.816–0.988)	<0.001
	24-R	737.0 ± 320.4	751.1 ± 346.5	0.988 (0.953–0.997)	<0.001
Zinc (mg/day)	DBBB	12.0 ± 3.6	12.8 ± 4.2	0.899 (0.618–0.974)	0.001
	24-R	12.4 ± 4.3	12.9 ± 6.1	0.921 (0.687–0.980)	0.001
Iodine (mg/day)	DBBB	142.3 ± 90.1	149.5 ± 83.6	0.988 (0.953–0.997)	<0.001
	24-R	154.7 ± 75.5	158.0 ± 78.2	0.989 (0.958–0.997)	<0.001
Calcium (mg/day)	DBBB	819.2 ± 220.3	862.7 ± 331.8	0.794 (0.158–0.949)	0.017
	24-R	840.7 ± 276.1	814.2 ± 323.4	0.969 (0.883–0.992)	<0.001
Vitamin D (µg/day)	DBBB	3.7 ± 1.9	4.0 ± 2.4	0.879 (0.511–0.970)	0.003
	24-R	4.7 ± 3.2	3.8 ± 2.9	0.883 (0.559–0.971)	0.001
Vitamin E (mg/day)	DBBB	9.7 ± 4.6	9.7 ± 4.5	0.851 (0.366–0.963)	0.006
	24-R	9.3 ± 3.9	12.1 ± 10.2	0.325 (−1.73–0.833)	0.287
Sodium (mg/day)	DBBB	2580.0 ± 894.9	2869.1 ± 1393.5	0.875 (0.532–0.968)	0.002
	24-R	2632.1 ± 1106.7	2524.1 ± 1042.9	0.976 (0.908–0.994)	<0.001
Potassium (mg/day)	DBBB	2724.0 ± 832.0	2543.2 ± 849.4	0.852 (0.435–0.963)	0.005
	24-R	2750.7 ± 844.8	2679.1 ± 816.1	0.961 (0.849–0.990)	<0.001
Magnesium (mg/day)	DBBB	301.4 ± 65.1	274.0 ± 77.7	0.741 (0.069–.934)	0.024
	24-R	297.8 ± 88.0	282.3 ± 86.3	0.959 (0.839–0.990)	<0.001

^1^ SD standard deviation; ^2^ ICC Intra-class Correlation Coefficient; ^3^ DBBB Analysis based on DietBytes image-based dietary records; ^4^ 24-R Analysis based on 24-h recall; ^5^ Folate as dietary folate equivalents.

**Table 4:** Quality assessment of the DietBytes image-based dietary record entries (*n* = 517 entries for *n* = 25 participants).

	Yes (*n*)	Yes (%)
Images		
Is there an image in the record?	496	95.94
*If yes, is the reference card visible?*	439	88.51
*If yes, can all food items be clearly seen?*	430	86.69
*If yes, is the image sufficient to quantify items?*	439	88.51
Voice Records		
Is there a voice record present?	158	30.56
*If yes, does the voice record include the item name?*	157	99.37
*If yes, does the voice record include the item type?*	117	74.05
*If yes, does the voice record include the item brand/product name?*	50	31.65
*If yes, does the voice record include item preparation /cooking methods?*	48	30.38
*If yes, is the voice record sufficient to identify items?*	140	88.61
Text Description		
Is there text description present?	312	60.35
*If yes, does the text description include the item name?*	307	98.40
*If yes, does the text description include the item type?*	177	56.73
*If yes, does the text description include the item brand/product name?*	83	26.60
*If yes, does the text description include preparation /cooking methods?*	50	16.03
*If yes, is the text description sufficient to identify the item?*	225	72.12
Is there an image and a voice record?	155	29.98
Is there an image and a text description?	297	57.45
Is there an image, voice record, and text description?	15	2.90

**Table 5:** Participants’ perceived usability and acceptability of using the Diet Bytes method for dietary assessment (*n* = 25).

Perceived Usability and Acceptability ^1^	Count (%)					
	Strongly Agree	Agree	Neutral		Disagree	Strongly Disagree
It was easy to use the Evernote app to collect my photographic dietary record	9 (36)	15 (60)	1 (4)		0 (0)	0 (0)
It was difficult to take photographs of my food and drinks	0 (0)	3 (12)	1 (4)		13 (52)	8 (32)
I found using the voice record annoying	4 (16)	4 (16)	8 (32)		7 (28)	2 (8)
I found it difficult to remember to collect a photographic dietary record	1 (4)	3 (12)	6 (24)		11 (44)	4 (16)
The text message reminders helped me to remember to use the app	4 (16)	17 (68)	3 (12)		0 (0)	1 (4)
I found the prompt card helpful	6 (24)	11 (44)	7 (28)		1 (4)	0 (0)
		**Yes *n* (%)**			**No *n* (%)**	
Did the way you used the app in private and in public differ?		15 (60)			10 (40)	
Did you record all food and drink items that you consumed during the period that you collected a photographic dietary record?		17 (68)			8 (32)	
Would you use the smartphone photographic dietary record method again?		22 (88)			5 (20) ^2^	
*If yes, would you want to use the photographic dietary record to do any of the following: n* (%)						
Share it with a Dietitian for feedback	18 (72)					
Share with friends	4 (16)					
For your own feedback or tracking of your diet	16 (64)					
*Did you prefer to record details of your food and drink using:*						
Text description; *n* (%)	20 (80)					
Voice record; *n* (%)	5 (20)					
As a result of collecting a photographic dietary record did you do any of the following:	Yes; *n* (%)			No; *n* (%)		
Change the types of food you ate	7 (28)			18 (72)		
Change how often you ate	6 (24)			19 (76)		
Change the amount of food you ate	3 (12)			22 (88)		
Change where you ate	2 (8)			23 (92)		
Change who you ate with	0 (0)			25 (100)		
Change your cooking habits	1 (4)			24 (96)		

^1^ Questions as posed to participants; ^2^ Two participants responded both yes and no for this question.
